# Structurally Designed Attenuated Subunit Vaccines for *S. aureus* LukS-PV and LukF-PV Confer Protection in a Mouse Bacteremia Model

**DOI:** 10.1371/journal.pone.0065384

**Published:** 2013-06-07

**Authors:** Hatice Karauzum, Rajan P. Adhikari, Jawad Sarwar, V. Sathya Devi, Laura Abaandou, Christian Haudenschild, Mahta Mahmoudieh, Atefeh R. Boroun, Hong Vu, Tam Nguyen, Kelly L. Warfield, Sergey Shulenin, M. Javad Aman

**Affiliations:** Integrated Biotherapeutics Inc., Gaithersburg, Maryland, United States of America; Indian Institute of Science, India

## Abstract

Previous efforts towards *S. aureus* vaccine development have largely focused on cell surface antigens to induce opsonophagocytic killing aimed at providing sterile immunity, a concept successfully applied to other Gram-positive pathogens such as *Streptococcus pneumoniae*. However, these approaches have largely failed, possibly in part due to the remarkable diversity of the staphylococcal virulence factors such as secreted immunosuppressive and tissue destructive toxins. *S. aureus* produces several pore-forming toxins including the single subunit alpha hemolysin as well as bicomponent leukotoxins such as Panton-Valentine leukocidin (PVL), gamma hemolysins (Hlg), and LukED. Here we report the generation of highly attenuated mutants of PVL subunits LukS-PV and LukF-PV that were rationally designed, based on an octameric structural model of the toxin, to be deficient in oligomerization. The attenuated subunit vaccines were highly immunogenic and showed significant protection in a mouse model of *S. aureus* USA300 sepsis. Protection against sepsis was also demonstrated by passive transfer of rabbit immunoglobulin raised against LukS-PV. Antibodies to LukS-PV inhibited the homologous oligomerization of LukS-PV with LukF-PV as well heterologous oligomerization with HlgB. Importantly, immune sera from mice vaccinated with the LukS mutant not only inhibited the PMN lytic activity produced by the PVL-positive USA300 but also blocked PMN lysis induced by supernatants of PVL-negative strains suggesting a broad protective activity towards other bicomponent toxins. These findings strongly support the novel concept of an anti-virulence, toxin-based vaccine intended for prevention of clinical *S. aureus* invasive disease, rather than achieving sterile immunity. Such a multivalent vaccine may include attenuated leukotoxins, alpha hemolysin, and superantigens.

## Introduction

*Staphylococcus aureus* (SA) is a ubiquitous, formidable Gram-positive pathogen associated with a wide range of pathologies from skin and soft tissue infections (SSTI) to life-threatening systemic infections. SA is a leading cause of hospital-associated (HA) and community-associated (CA) infections worldwide [1], [2], [3], [4]. The range of pathologies reflects the diverse abilities of this microbe to escape the innate and adaptive immune responses using multiple virulence factors including coagulases, capsular polysaccharides, adhesins, proteases, exoproteins that inactivate the complement system, pore-forming toxins, superantigens and other innate response mediators [1], [5]. The rapid spread of methicillin resistant SA (MRSA) underscores the importance of developing vaccines for prevention or reduction of severity of MRSA infections. Most previous approaches for vaccine development have focused on achieving sterile immunity and largely ignored the potential for an anti-virulence approach aimed at clinical protection against invasive disease. Toward this goal, key secreted toxins of *S. aureus* such as superantigens and pore-forming toxins represent excellent vaccine targets.

While MRSA strains were initially limited to health care settings, since 1990s several epidemics of community associated *S. aureus* (CA-MRSA) have been reported that cause severe disease in otherwise healthy population. To date, five CA-MRSA clonal lineages have been associated with these outbreaks: the Pandemic clone (USA300, CC8), the Midwest clone (USA400, CC1), the European clone (CC80), the Southwest-Pacific Oceania clone (CC30), and the Pacific clone (CC59) [6]. In addition to SCC*mec* IV, a characteristic feature of these major CA-MRSA lineages is that they all have the *luk*PV operon encoding the Panton Valentine Leukocidin (PVL) [6], carried by the lysogenic phages φSLT, φPVL, φSA2MW and φSA2usa [7], [8], [9]. PVL positive *S. aureus* infections often affects young adults that had neither recent contacts with health care facilities nor any major risk factors and typically leads to high mortality rates [10], [11].

The pore forming toxins, consisting of single-component alpha-hemolysin and the bi-component hemolysins and leukotoxins, play an important role in staphylococcal immune evasion. These toxins kill key immune cells and cause tissue destruction, thereby often weakening the host during the first stage of infection and promoting bacterial dissemination and metastatic growth in distant organs. The two PVL components LukS-PV and LukF-PV are secreted separately, and form the pore-forming octameric complex upon binding of LukS-PV to its receptor and subsequent binding of LukF-PV to LukS-PV [12], [13]. Targets of PVL are polymorphonuclear phagocytes (PMN), monocytes, and macrophages. PVL is closely related to other bicomponent toxins including S components HlgA and HlgC and the F component HlgB of γ-hemolysin; LukE (S) and LukD (F); and LukM (S) and LukF-PV-like (F) [14]. Due to their close similarity any of these S components can combine with any F component and form an active new toxin with similar or modified target specificity [15], [16]. While the leukocidins primarily lyse neutrophils, Hlg is able to lyse both red blood cells [14] and neutrophils [17]. It has been reported that pairing of HlgA or HlgC with LukF-PV promotes the leukotoxic activity of Hlg [16]. Due to these similarities it is conceivable that vaccine-induced neutralizing antibodies towards PVL subunits may provide protection against other members of bicomponent toxins.

Due to pairing possibilities a wild type single subunit leukocidin or Hlg vaccine is not considered safe. Therefore, we sought to identify attenuating mutations in LukS-PV and LukF-PV. Beside the improved safety profile, attenuated subunits allow that both S and F subunits be used in combination, should this be required. Oligomerization of the S and F subunits is a pre-requisite for pore formation. We have recently published an all-atom model for LukS/LukF-PV dimers and octamers [18]. Using this model, we designed point mutations aimed at disrupting the oligomerization. Here we report successful identification of two highly attenuated LukS-PV and LukF-PV mutants capable of protecting from lethal bacteremia with the CA-MRSA USA300 in mice. Furthermore, antibodies against LukS-PV were shown to cross protect against leukotoxic activities of Hlg as well as supernatants from PVL-negative strains.

## Materials and Methods

### Bacterial Strain and Inoculum Preparation

*S. aureus* strain USA300 (NRS 384) and NCTC8325-4 (NRS77) were obtained from the NARSA repository. For bacterial challenges, CA-MRSA USA300 was grown for 18 hours in Tryptic soy broth (TSB, Difco Laboratories, Detroit, Mich.). 10 ml of TSB in 25 ml flask was inoculated with a single bead of *S. aureus* USA300 from −80°C stored bead stock and culture grown overnight at 37°C, with shaking at 230 rpm. The culture was centrifuged at 3000 rpm at RT, washed once with PBS and the bacterial pellet re-suspended in 1 ml sterile PBS and used for challenges as described below.

### Animals

Female BALB/c mice, 6 weeks of age for active immunogenicity studies and 10–12 weeks for passive vaccination studies, were purchased from Charles River laboratories. Mice were maintained under pathogen-free conditions and fed laboratory chow and water *ad libitum*. All mouse work was conducted in accordance with protocols approved by institutional animal care and use committees (IACUC) of Nobel Life Sciences (Gaithersburg, MD 20878), where animal studies were performed.

### Immunizations

For active immunogenicity studies, mice were immunized intramuscularly (IM) three times at two-weeks interval with the various antigens formulated in adjuvant. The doses of vaccine and adjuvant are specified for each experiment in the results section. For immunization with Al(OH)_3_ or AlPO_4_ the antigen was pre-absorbed to adjuvant for 1 hour at a ratio of 1∶7 (antigen:adjuvant) in 50 mM Tris, pH 7.5. GLA-SE or CPG were mixed with the antigen in PBS before injection. To determine serum titer concentrations, mice were bled prior to first and 10 days post last immunization. For passive immunization studies, mice were treated with 250 µg of rabbit polyclonal anti-LukS-PV antibodies or control rabbit naïve IgG in 500 µl volume of PBS via intraperitoneal (IP) administration 24 hours prior to bacterial challenge.

### Mouse Bacteremia Model

Female BALB/c mice were challenged via IP injection with CA-MRSA USA300 in 3% mucin-PBS solution as previously described [19]. Briefly, lyophilized hog mucin type III (Sigma Aldrich, St. Louis, Mo) was solubilized to 6% (w/v) in PBS, sterilized by autoclaving for 10 minutes and rapidly cooled on ice for 10–15 minutes. For bacterial challenges, overnight culture of USA300 was washed twice and re-suspended in PBS to an optical density of 0.15 at 600 nm, corresponding to 7×10^7^ CFU/ml, and then adjusted to desired concentrations with PBS. At the time of challenge, bacteria and mucin solution were mixed at equal volumes and mice injected IP with 0.5 ml containing the desired inoculum dose in 3% mucin-PBS. Mice were monitored for morbidity and mortality twice a day up to 15 days post challenge. To determine bacterial dissemination to organs, mice were euthanized 3 days after challenge and blood and organs (liver, combined kidneys, lungs and spleen) were aseptically removed. Organs were homogenized with sterile 3.2 mm stainless steel beads using a Bullet Blender from Next Advance Inc. (Averill Park, NY) and were taken up in a total volume of 500 µl PBS. Blood samples and organ homogenates were streaked out in different dilutions on Brain Heart Infusion (BHI, Becton Dickinson) agar plates and CFU was enumerated after ON incubation at 37°C.

### Enzyme-linked Immunosorbant Assay (ELISA) for Determination of Serum Titers

Blood samples from mice were centrifuged in serum separator tubes and serum samples were stored at −80°C until further use in ELISA. Briefly, 96-well plates were coated with 100 ng/well of His-tagged wild type (wt) LukS-PV, wt LukF-PV proteins, or staphylococcal enterotoxin B (SEB), overnight at 4°C. Plates were blocked with Starting Block buffer (Thermo Scientific) for one hour at room temperature (RT). Serum samples were prepared in semi-log dilutions starting from 1∶100 to 1∶10,000.000 in a 96-well plate using starting block buffer as diluent. Plates were washed three times and sample dilutions were applied in 100 µl volume/well. Plates were incubated for one hour at room temperature (RT) and washed three times before applying the conjugate, goat anti-mouse IgG (H&L)-HRP (Horse Radish Peroxidase) in starting block buffer. Plates were incubated for one hour at RT, washed as described above and incubated with TMB (3,3′,5,5′-tetramethylbenzidine) to detect HRP activity for 30 min. Optical density at 650 nm was measured using a Versamax™ plate reader (Molecular Devices, CA). Data analysis for full dilution curves was performed using Softmax program.

### Plasmids, Protein Expression and Purification

Wild type PVL and HlgB/C subunits were produced by gene synthesis and cloned into pQE30 vector with an N-terminal 6xHis tag for purification. Site-directed mutagenesis was carried out using QuikChange II Site-Directed Mutagenesis Kit (Stratagene, La Jolla, CA) as per the manufacturer’s instructions. For LukS-PV and LukF-PV single mutants, we introduced a single amino acid change in site-directed mutagenesis primers. For introducing double and triple mutations, single mutant was used as template to introduce double mutations and double mutant as template to introduce triple mutation. His-tagged wild type subunits were generated.

Proteins were produced in *E. co*li XL1-Blue and purified using HisTrap HP column using a protocol that we previously described [20]. All proteins showed the expected molecular weight and over 95% purity. Pure proteins were dialyzed overnight in PBS supplemented with 10% Glycerol, pH 7.4 as the storage buffer. Dialyzed proteins were further concentrated using Amicon 3 K filters (Millipore) and sterile filtered by 0.22 µM syringe filters (Millipore). Finally, the concentration of proteins was determined by bicinchoninic acid (BCA) assay. SDS-PAGE and Western blot analysis were performed using rabbit polyclonal antibodies raised against the respective antigens. The proteins were stored at –80°C in aliquots until they were used for further studies. The endotoxin levels in protein samples were determined by Limulus Amoebocyte Lysate (LAL) chromogenic endotoxin assay as described in our previous report [20].

### Thermo Fluor Assay and Data Analysis

Sypro Orange purchased from Invitrogen (Carlsbad, CA) is commercially supplied as 5000× concentrated in dimethyl sulfoxide (DMSO) solution. The concentration of the PVL mutant proteins used for this assay was 0.1 mg/mL. The dye was added to the protein to achieve a final 1× concentration immediately prior to the fluorescence measurement. 100 uL of the protein/dye solution was transferred to the well of a Corning 96-well black flat bottom plate. The plate was heated from 25 to 95°C at 1°C/minute heating rate on the Tecan infinite M200 plate reader and the fluorescence emission scan was read for every 5°C. Fluorescence measurements were taken using the excitation wavelength 490 nm and the emission signal was monitored from 520 to 700 nm at 120 nm/min scanning speed. The corrected protein spectra after subtracting the buffer/dye baseline were used for data analysis and all the spectra were the results from the average of three measurements. Protein spectra were plotted and analyzed using Origin 7.0 (Origin Lab, Northampton, MA) software. The maximum fluorescence emission intensity observed at 588 nm was plotted at each temperature for the proteins. The inflection point of the transition curve (T_m_) was calculated using Boltzmann Sigmoid fit. For the denaturation curves, two state unfolding was assumed between 25°C to 80°C and the fraction unfolded was calculated according to the literature [21].

### Molecular Modeling

Model building, computer calculations, and structural analysis were performed using the Accelrys, Inc software Discovery Studio 2.5 running on a Dell Precision 690 running Red Hat Enterprise Linux 4. Simulations were *in vacuo* and employed a CHARMM force field with a CFF partial charge, a distance-dependent electric constant of 1, and a temperature of 300 K. Energy minimization involved 1000 steps of conjugate gradient. A complex consisting of LukF-PV and LukS-PV was extracted from our previously described model of the LukF-LukS octamer [18] for this study. Two LukF-LukS dimer structures were extracted to represent the two different types of protein-protein interfaces represented in the PVL octamer model structure. These two dimer structures were termed the F_R_-S_L_ and S_R_-F_L_ interface models. The LukF-PV/LukS-PV dimer structures were energy minimized to relax the structure. Amino acids at the protein-protein interfaces were mutated, and the dimers were energy minimized with polypeptide backbone atoms fixed in Cartesian space. To quantify the effect of unfavorable contacts due to mutations, molecular energies of dimers were calculated using Discovery Studio and compared to the wild-type dimer structures.

### MPD Oligomerization Assay

Oligomerization assay for PVL and Hlg subunits was performed based on a previously described protocol [22] using 2-methyl-2,4-pentanediol (MPD). Equal amounts 30 µg/ml of each protein component were incubated together in the presence of 40% MPD for 24 hours at RT in 31.25 µl total reaction volume. In certain experiments the incubation time was reduced to 1 h and/or the incubation performed at various temperatures. In one experiment, 100 µg/ml of each toxin components were also tested. 15 µl of the mixture was electrophoresed (without boiling) in the presence of 1× Laemmli’s SDS sample buffer on a 4–20% polyacrylamide gel. Gels were stained with Gel Code Blue™ reagent to visualize the bands.

### Inhibition of Subunit Oligomerization by Rabbit Anti-LukS Antibodies

A constant concentration of LukS-PV (30 µg/ml) was incubated with decreasing concentrations of anti-LukS polyclonal antibodies (1100-17.2 µg/ml) at 2-fold serial dilutions) for 30 minutes at RT. An equivalent concentration of homologous (LukF-PV) or heterologous (HlgB) subunit and MPD (final concentration of 40%) was added and mixture was incubated at room temperature for 24 hours. 15 µl samples were taken and analyzed by SDS-PAGE. Gels were stained with Gel Code Blue™ reagent to visualize the bands. Naïve rabbit IgG was used as negative control.

### HL-60 Based Cytotoxicity and Neutralization Assay for Leukocidin

PVL activity was determined based on its cytotoxicity in differentiated HL-60 cells (ATCC, Manassas, VA). The HL-60 cells were propagated for seven days prior to the assay in RPMI media supplemented with 15% fetal bovine serum (FBS) and 1.5% dimethylsulfoxide (DMSO). The differentiated neutrophil-like cells were harvested and suspended in phenol red free RPMI/2% FBS for cytototoxicity assay. The final density of 5×10^5^ cells/well were incubated either with various concentrations of PVL (LukS-PV+LukF-PV), gamma hemolysin subunits (HlgB+C) or heterologous combinations of the subunits from PVL and gamma hemolysins for 48 hours at 37°C as described in our previous report [23]. For neutralization experiments, pooled mouse sera or purified rabbit IgG were first serially diluted in duplicates in V-bottom polypropylene 96-well plates. PVL (1∶1 LukS-PV and LukF-PV) or gamma hemolysin (1∶1 HlgB and HlgC) were added at 750 ng/ml for each subunit. Alternatively bacterial supernatants were used as source of leukotoxin. In this case 0.2 µm-filtered supernatants of overnight cultures of CA-MRSA USA300 or *S. aureus* 8325-4 were added to the wells. Samples were then transferred to 96-well round bottom culture plates containing HL-60 derived neutrophils at a final density of 5×10^5^ cells/well, mixed and incubated for 48 hours at 37°C in an atmosphere of 5% CO_2_-95% air. The cellular viability was evaluated after 16 hours of further incubation with 100 µg/ml of XTT (Sigma-Aldrich, St. Louis MO) and colorimetric measurement at OD_470_ nm, from which neutralization titers (NT_50_) were determined for each sample [23].

## Results

### Design of PVL Mutants

**Figure 1:**
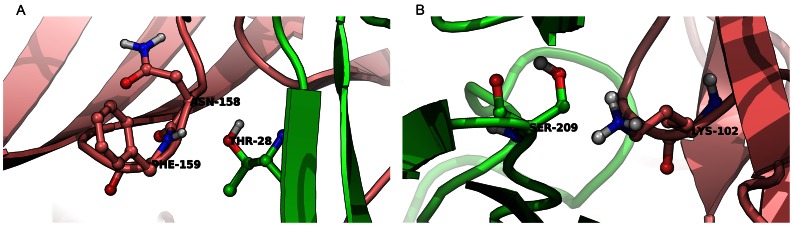
Key residues involved in interaction of LukS-PV and LukF-PV. ***A)*** Interface interactions between T28 of LukS-PV (green ribbon) and N158 and F159 of LukF-PV (pink ribbon). ***B)*** Interface interaction between S209 of LukS-PV (green ribbon) and K102 of LukF-PV (pink ribbon).

To develop a vaccine that is composed of attenuated forms of LukS-PV and/or LukF-PV, we sought to modify the subunits to disrupt oligomerization, prevent *in vivo* pore assembly, and the subsequent cytolysis while maintaining the overall structural integrity and immunogenicity. We have recently generated an all-atom model of the pre-pore conformation of LukF-PV/LukS-PV heterodimer and octamer [18] which was used in the current study for rational design of attenuated PVL mutants. This model delineates a structural basis for previously reported results from mutagenesis studies by providing models of the two distinct protein-protein binding surfaces found in oligomeric LukF-PV/LukS-PV, which we termed the F_R_-S_L_ and S_R_-F_L_ interface models [18]. For example, Guillet *et al* had found that the LukS-PV T28S mutant was as active as wild-type, whereas the T28H and T28C mutants were slightly less active than wild-type, and lastly, the T28L, T28F, T28N, and T28D mutants were largely inactive [24]. Because T28 is a surface residue, the 1T5R monomeric crystal structure of LukS-PV [24] does not fully provide a structural basis for these experimental results. In contrast, our F_R_-S_L_ model [18] shows that the T28 side chain is tightly packed against the polypeptide backbone of residues N158 and F159 in the neighboring LukF-PV subunit (***Figure 1A***). To illustrate this, the T28 OG atom is ∼3.5 Å away from both N158 CA and F159 NH of LukF-PV (***Figure 1A***), which are residues in a loop structure. This tight packing explains why sterically bulkier T28L, T28F, T28N, and T28D mutants are attenuated, whereas the conservative T28S mutant was as active as wild-type.

To identify other interaction sites that may be crucial to oligomerization, we used molecular modeling to scan the F_R_-S_L_ and S_R_-F_L_ interfaces in our PVL octamer model for hotspots that if mutated, would significantly shift the monomer-dimer equilibrium constant in favor of the monomer. In LukS-PV, these additional sites are Y131, and S209, which were identified from the F_R_-S_L_ interface model and K97 and D101 from the S_R_-F_L_ model [18]. The corresponding sites in LukF-PV were K102 and D121 in the F_R_-S_L_ model and E147 and N220 in S_R_-F_L_ model.

As one of the metrics used to determine the effect of each mutant on dimerization, a series of positions were mutated to alanine *in silico* for residues outside of LukS-PV T28 and to phenylalanine for T28. Because the LukS-PV S209 hydroxyl group is hydrogen bonded to the K102 side chain amino group in LukF-PV, its alanine mutation results in the loss of a critical hydrogen bond that ties together the short 207–209 loop of LukS-PV with the 101–107 loop structure in LukF-PV [18] (***Figure 1B***). Given that single point mutations at these sites are predicted to shift the monomer-heterodimer equilibrium of PVL in favor of monomers, these mutants are candidates for vaccine development.

Because T28 on LukS-PV has been reported to play a role in dimerization, we examined the potential utility of T28 in double mutants. In this analysis, the double mutants T28F/Y131A, T28F/S209A, as well as a triple mutant consisting of T28F/Y131A/S209A were investigated *in silico*. Upon calculation of molecular energies for each construct the mutants listed in ***Table 1*** were selected for further investigation.

**Table 1:** List of LukS-PV and LukF-PV mutants showing increased molecular energy in the heterodimer model.

LukS-PV Mutants	Mutation
Mut2	T28F
Mut3	K97A
Mut4	D101A
Mut5	Y131A
Mut6	S209A
Mut7	T28F/Y131A
Mut8	T28F/S209A
Mut9	T28F/K97A/S209A
**LukF-PV Mutants**	**Mutation**
Mut1	K102A
Mut2	D121A
Mut3	E147A

### Generation and Production Of Mutants

**Figure 2:**
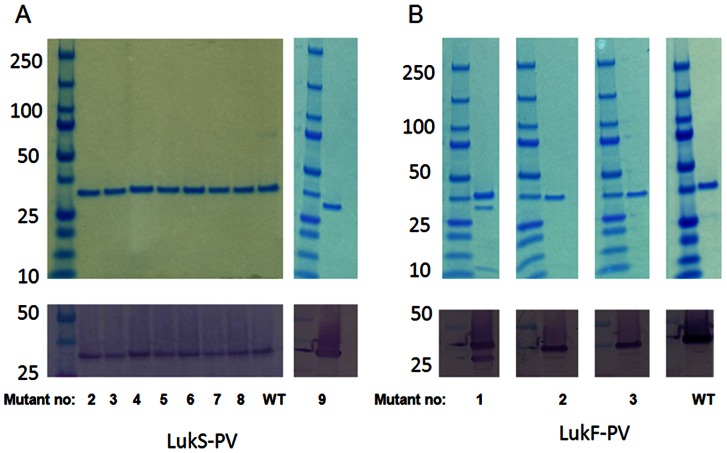
SDS-PAGE and Western blot of mutant and wild type forms of LukS-PV and LukF-PV. ***A)*** LukS-PV wt and mutants 2–9, ***B)*** LukF-PV wt and mutants 1–3. Upper panels: coomassie staining; Lower panels: Western blot.

Mutants listed in ***Table 1*** were generated by site directed mutagenesis in pQE30 vector with an N-terminal 6xHis tag for purification. Similarly His-tagged wild type subunits were generated. Proteins were produced in *E. coli* XL1-Blue cells and purified using HisTrap HP column. All proteins showed the expected molecular weight and over 95% purity as shown on SDS-PAGE and Western blot analysis for LukS-PV Mut2–9 (***Figure 2A***) and for LukF-PV Mut1–3 (***Figure 2B***).

### *In vitro* Structural and Functional Characterization of Mutant Proteins

**Figure 3:**
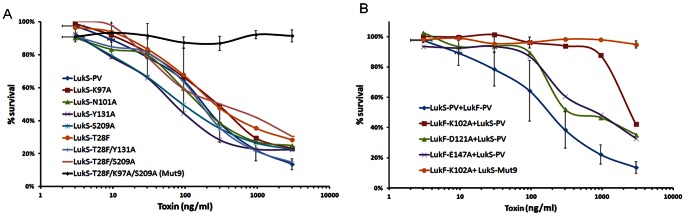
Attenuation of PMN lytic activity of PVL mutants. ***A)*** % survival of HL-60 derived neutrophils treated with increasing concentrations of wt or mutant LukS-PV each in combination with wt LukF-PV. Results represent mean values from 5 independent experiments. STDV is shown only for wt and the triple mutant (LukS-Mut9). ***B)*** % survival of HL-60 derived neutrophils treated with increasing concentrations of wt or mutant LukF-PV each in combination with wt LukS-PV or LukS-PV triple mutant (LukS-Mut9).

Functional activity of the mutants was tested in a cytotoxicity assay using HL-60 cells differentiated to neutrophils as previously described for opsonophagocytosis assays [25]. As shown in ***Figure 3A***, single mutants of LukS-PV did not have a significant impact on PVL toxicity when the mutants were combined with wild type (wt) LukF-PV, and double mutants only slightly reduced the PVL toxicity. However, the triple mutant LukS-PV_T28F/K97A/S209A (denoted hereafter as LukS-Mut9) was completely attenuated when combined with wt LukF-PV (***Figure 3A***). When combined with wt LukS-PV, all three single mutants of LukF-PV showed a reduction in toxicity demonstrated by the right shift in dose response curve (***Figure 3B***). The highest attenuation was achieved with LukF-PV_K102A (denoted hereafter as LukF-Mut1) (***Figure 3B***). Residual toxicity of LukF-PV_K102A was seen only at concentrations above 1000 ng/ml. As expected, when this mutant was combined with LukS-PV Mut9, no toxicity was observed (***Figure 3B***).

### Thermal Stability Analysis

**Figure 4:**
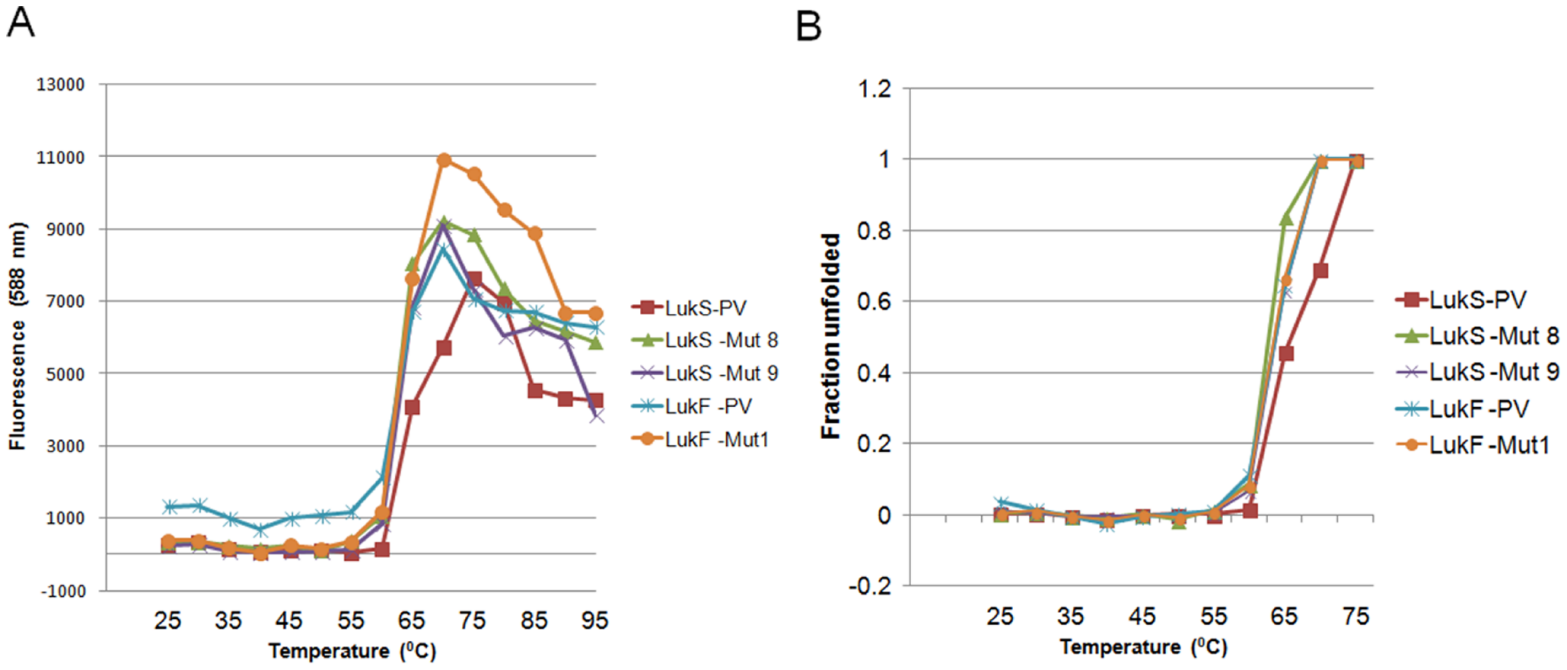
Thermal unfolding of LukS-PV and LukF-PV proteins as monitored by thermofluor assay using Sypro Orange dye. *****A)***** Plot of fluorescence intensity of PVL proteins at 588 nm against temperature. Data was collected for every 5°C. ***B)*** Plot of fraction unfolded calculated from the thermal denaturation curve. Key: LukS-PV wild type, LukS-PV T28F/S209A (LukS-Mut8), LukS-PV T28F/K97A/S209A (LukS-Mut9), LukF-PV Wild type and LukF-PV K102A (LukF-Mut1) were tested.

Thermal stability of LukS-PV and LukF-PV mutant proteins was assessed by Thermofluor (Differential Scanning Fluorimetry) using Sypro Orange as the external fluorescent probe which binds to hydrophobic residues detecting their exposure during protein unfolding. Heat induced protein unfolding will therefore usually result in increased fluorescence. However, if unfolding leads to aggregation the result is a decreased florescence. This increase and decrease of the fluorescent signal is an excellent means to monitor protein unfolding, calculate the melting temperatures, and compare the thermal stabilities of different proteins under different experimental conditions [26], [27]. ***Figure 4A*** shows the changes in fluorescent signal of the proteins during thermal unfolding in the presence of the dye while ***Figure 4B*** is the plot of fraction unfolded based on fitting each protein melting curve using the two-state equations [21]. Wild type and mutant proteins of both subunits showed very low background fluorescence at 25°C and retained the intensity until 55°C showing that the proteins are stable within this temperature range. Heating above 55°C caused an increase in the fluorescent signal indicating protein unfolding. This steep increase also supports a highly co-operative unfolding process. LukS-PV wt curve (black) is only slightly shifted to the right indicating that it is slightly more stable than the mutants at higher temperatures. The maximum fluorescence intensity was observed at 75°C for the wt LukS-PV while it was at 70°C for wt LukF-PV as well as all mutants tested. When heated above those temperatures, the fluorescence intensity dropped for all the proteins indicating an aggregation event taking place. Therefore, intensity values only up to 75°C were considered for creating the fraction unfolded plot (***Figure 4B***). Apparent melting temperature (T_m_) values from the Boltzmann Sigmoid fitting of the data showed that the T_m_ for all the mutants ranged from 62.6 to 63.6°C similar to wt LukS-PV (64.8°C) and wt LukF-PV (62.9°C) suggesting that introduced mutations did not significantly affect the thermal stability of proteins.

### Impact of Mutations on Leukocidin Oligomerization

**Figure 5:**
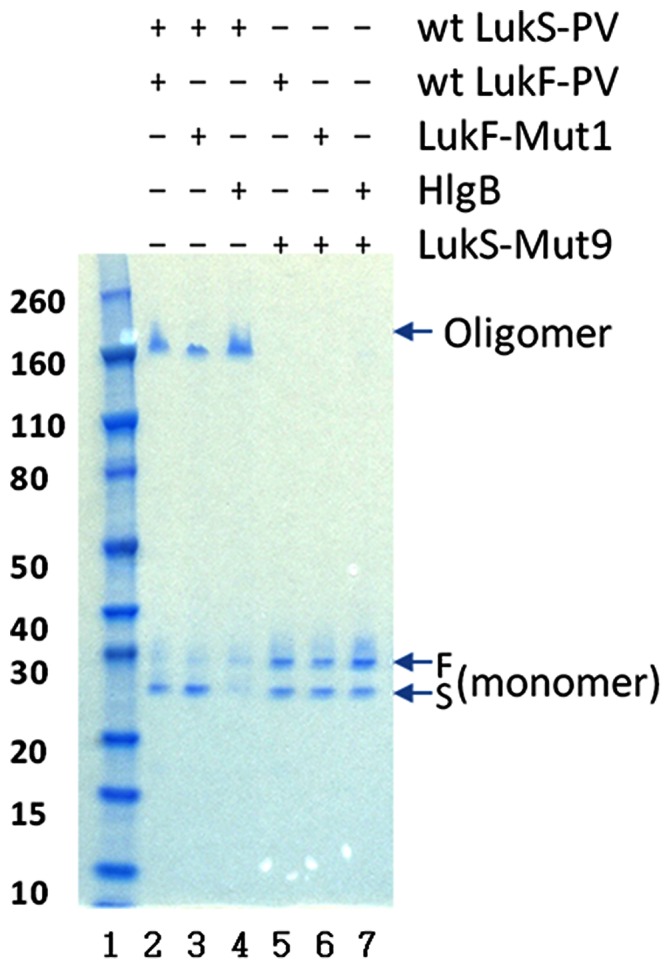
Homologous and heterologous oligomerization of wt and mutant LukS-PV and LukF-PV. Individual subunits as indicated above the panel were co-incubated overnight at a concentration of 30 µg/ml with 40%MPD and analyzed by SDS-PAGE without boiling. Lane 1: molecular weight Marker; Lanes 2–5: different combinations of wild type (wt) or mutant leukocidins as shown above the panel.

**Figure 6:**
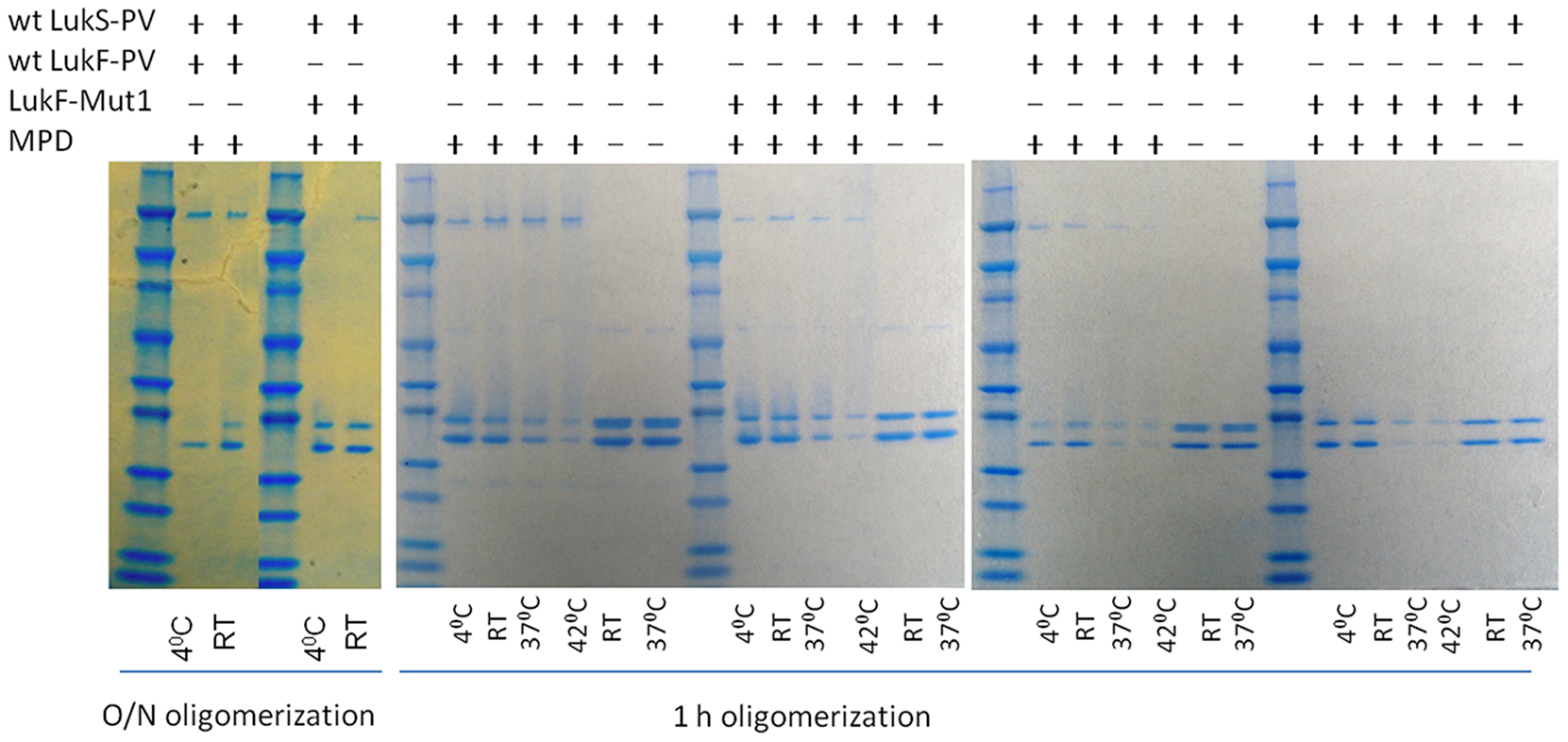
LukF-Mut1 displays reduced oligomerization activity. Individual subunits as indicated above the panel were co-incubated with or without MPD at a concentration of 30 µg/ml(Left and right panels) or 100 µg/ml (middle panel) at indicated temperatures and analyzed by SDS-PAGE without boiling. Incubation time was either 24 hours (left panel) or 1 hour (middle and right panels). RT: room temperature.

Oligomerization of the leukocidin components is a required step for cytotoxicity of these toxins. PVL components have been shown to spontaneously form a pore structure identical to that in the biological membranes in presence of 2-methyl-2,4-pentanediol (MPD) [11]. We used this assay to test if the introduced mutations resulted in loss or reduction of PVL oligomerization. Furthermore, we tested the ability of the LukS-PV and LukS-Mut9 to form heterologous oligomers with gamma hemolysin B subunit (HlgB). As shown in ***Figure 5*** ***(Lane 2)***, wild type forms of LukS-PV and LukF-PV formed oligomers when incubated overnight at RT in presence of MPD. A similar oligomeric band was seen with LukF-Mut1 in combination with wild type LukS-PV (***Figure 5*** **,** ***Lane 3***) suggesting that LukF- Mut1, despite being attenuated, is still able to form oligomers with wt LukS-PV. Similarly, wt LukS-PV was able to form hetero-oligomers with HlgB (Lane 4). However, LukS-mut9 was unable to form oligomers with either wt LukF-PV (Lane 5), LukF-Mut1 (Lane 6), or HlgB (Lane 7). Since, despite our molecular model prediction and the functional data, LukF-Mut1 was able to form oligomers with wt LukS-PV, we hypothesized that the attenuation of LukF-Mut1 may have led to reduced stability of the oligomer rather than its complete abrogation. Hence, we sought to compare the oligomerization of the wild type and mutant LukF-PV under different incubation periods and at different incubation temperatures. We first compared the ability of the LukF-Mut1 and LukF-PV to form oligomers with wt LukS-PV after overnight incubation at 4°C versus room temperature (RT). As shown in ***Figure 6*** ***(left panel*** **),** while LukF-Mut1 readily forms oligomers at RT similar to the wild type, it poorly oligomerizes with wt LukS-PV at 4°C suggesting a slower kinetics resulting from the mutation. We then tested the oligomerization after 1h incubation at various temperatures. While native S and F subunits formed oligomers at temperatures as high as 42°C, the ability of LukF-Mut1 to oligomerize with wt LukS-PV was significantly reduced particularly at 37°C and 42°C (***Figure 6*** **,** ***middle panel***). This difference was more pronounced when the subunits were co-incubated at a lower concentration (30 µg/ml vs. 100 µg/ml) (***Figure 6, right panel***). These data suggest that the ability of LukF-Mut1 to oligomerize is reduced compared to the wild type counterpart.

### Immunogenicity and Adjuvant Studies

**Figure 7:**
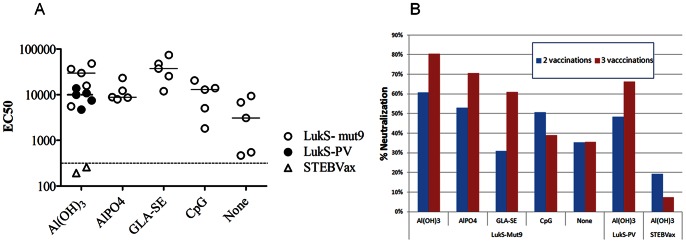
Immunogenicity of LukS-Mut9 with different adjuvants in mice. ***A***) Total Ab titers determined by ELISA for individual mouse sera (EC50; i.e. dilution of serum with 50% maximal signal on ELISA plates coated with wild type LukS-PV). ***B***) Neutralization determined in HL-60 toxin neutralization assay using wild type LukS-PV and LukF-PV toxins. Percent neutralization of wild type toxin is shown at 1∶100 dilution of serum from vaccinated mice (sera pooled from 5 mice in each group). Doses used: antigens: 10 ug; Al(OH)3∶34 µg, AlPO4∶70 µg, GLA-SE: 20 ug, and CpG: 10 µg/mouse.

An immunogenicity study was performed in mice using different clinically relevant adjuvants including two forms of alum: Alhydrogel (Al(OH)_3_) and aluminum phosphate (AlPO_4_), as well as two novel adjuvants currently in clinical trials: GLA-SE (glucopyranosyl lipid adjuvant stable emulsion) [28], [29] and CpG [30]. Groups of 5 female BALB/c mice were vaccinated intramuscularly (IM) three times with 5 µg of LukS-Mut9 with each of the adjuvants at 2 week intervals. As controls, we used the wild type (wt) LukS-PV as well as an unrelated antigen (STEBVax; staphylococcal enterotoxin B vaccine) combined with Alhydrogel. Mice were bled on days 21 and 35. All adjuvants induced robust total antibody response over one log higher than the no adjuvant control (***Figure 7A***). Neutralizing antibody titer was determined using HL-60 derived neutrophils as described above. As shown in ***Figure 7B***, highest neutralizing titer was achieved after three vaccinations using the alum-based adjuvants and GLA-SE. The antibody response to LukS-Mut9 was comparable to the response to wt LukS-PV suggesting the conservation of immunological epitopes in the mutant. Based on these results GLA-SE was selected for subsequent immunogenicity and efficacy studies.

### Efficacy Studies in Mice

**Figure 8:**
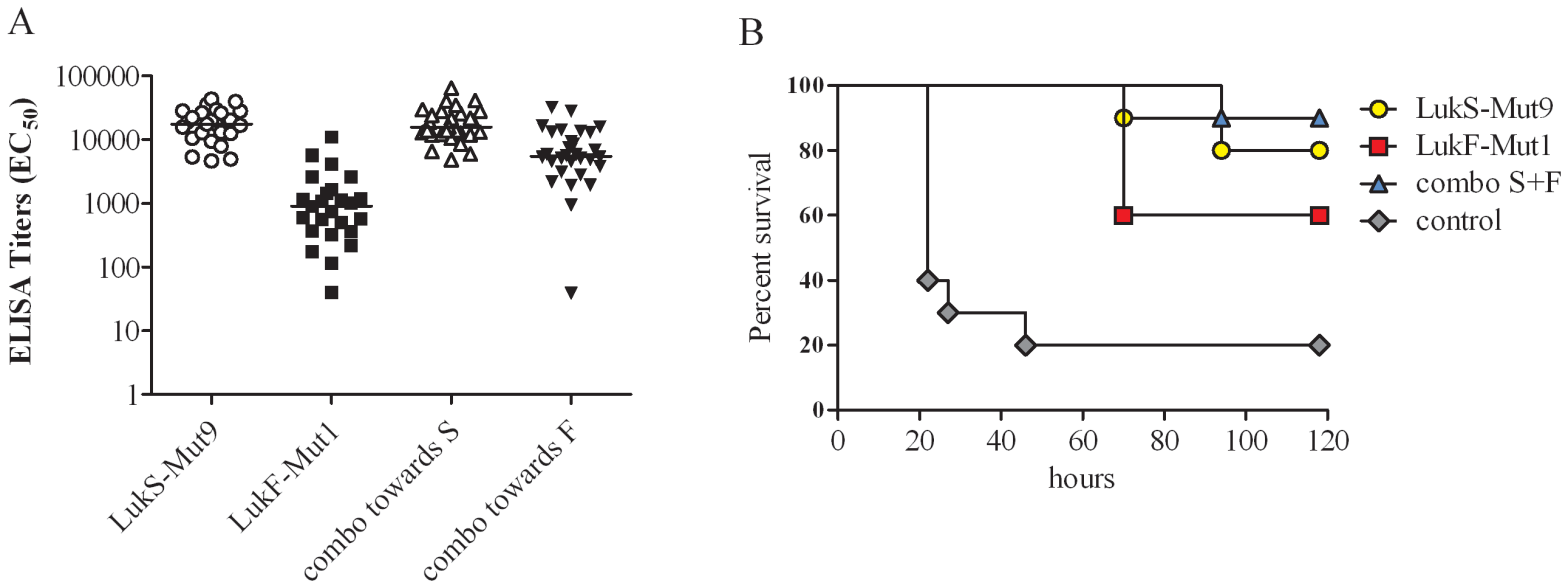
Imunogenicity and protective efficacy of LukS-Mut9 and LukF-Mut1. ***A)*** Antibody titers of mice immunized with lukS-Mut9 or LukF-Mut1 towards the homologous wild type antigens or the combination of both mutants towards each antigen. ***B)*** Protection from lethal challenge by active immunization with LukS-Mut9 and LukF-Mut1 along with GLA-SE in USA300 bacteremia model.

**Figure 9:**
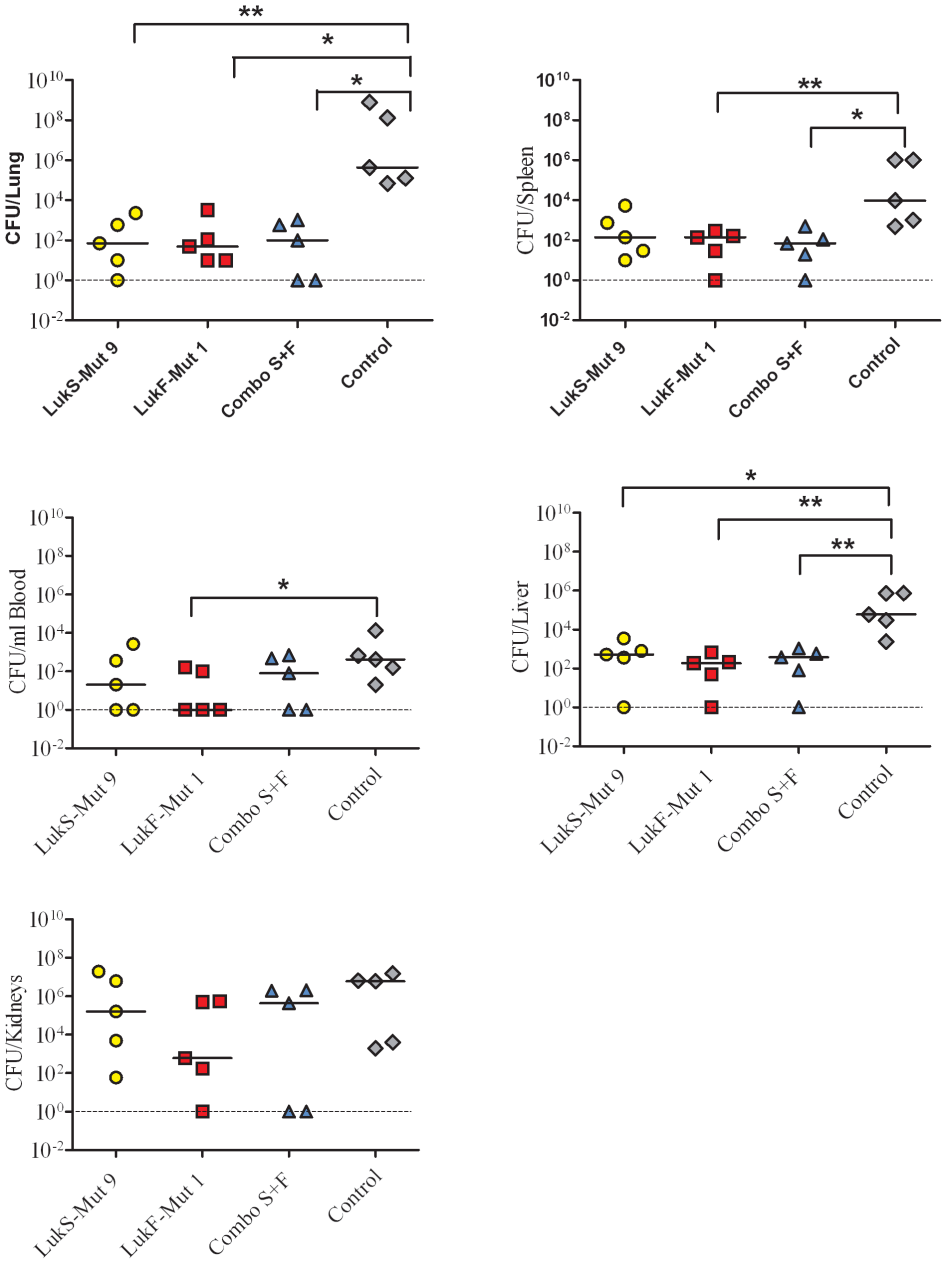
Effect of active immunization with PVL mutants on bacterial load. Bacterial loads were determined in blood and various organs of immunized mice 3 days post challenge with a sub-lethal dose of USA300.

Multiple efficacy studies were performed independently in a murine intraperitoneal sepsis model [19]. In these studies groups of 5–10 BALB/c mice were immunized intramuscularly three times at 2 week intervals with 10 µg of LukS-Mut9, LukF-PV-Mut1, combinations of both mutants, or BSA as control formulated with 20 µg of GLA-SE. Serum antibody titers post third immunization were tested by ELISA. ***Figure 8A*** summarizes the individual antibody titers against each antigen compiled from these studies. LukS-Mut9 induced a log higher Ab response than LukF-Mut1 towards respective wild type antigens. When serum titers of mice vaccinated with combination of both mutants were tested against wt LukS-PV or wt LukF- PV, titers were similar to LukS-Mut9 only immunized mice. ***Figure 8B*** shows a representative challenge study. Mice were challenged on day 42 with 5×10^4^ CFU of USA300 (∼LD_90_) in 3% hog mucin and monitored for 5 days post infection. Vaccination with LukF-Mut1 resulted in 60% protection while 8 out of 10 mice vaccinated with LukS-Mut9 and 9 out of 10 mice vaccinated with both mutants survived the challenge. In contrast, only 20% of mock-immunized mice survived. These data indicate that each mutant is able to contribute to protection. To determine bacterial dissemination to organs, additional groups of five vaccinated mice were challenged with a sublethal dose of USA300 (1.5×10^4^ CFU) and euthanized 3 days after bacterial infection. Blood and organs were aseptically removed, homogenized, and plated at different dilutions. As shown in ***Figure 9*** bacterial load was generally higher in mock-immunized mice with statistically significant differences observed in the lung, liver, and spleen.

To further examine the role of PVL antibodies in protection against sepsis, we performed a passive immunization study using rabbit polyclonal antibodies raised against LukS-PV (LukS-PV IgG). Groups of 10 BALB/c mice were injected IP with 250 µg of LukS-PV IgG or naïve rabbit IgG and challenged 24 h later IP with different doses of USA300. As shown in ***Table 2***, naïve IgG treated mice showed 30%, 20%, 10%, or 0% survival when challenged with 4.3×10^4^, 5.8×10^4^, 9.4×10^4^, or 2.5×10^5^ CFU of USA300, respectively. In contrast, LukS-PV IgG treated mice were fully protected from lethal challenge with up to 5.8×10^4^ CFU, and showed 80% survival at 9.4×10^4^ CFU, and 60% survival at 2.5×10^5^ CFU. These data suggest that protection against USA300 sepsis observed in active vaccination is primarily mediated by antibodies.

**Table 2:** Passive immunization with rabbit anti-LukS-PV antibodies protects against lethal challenge with USA 300. Fractions indicate survivors/total number of mice.

Challenge (CFU)	Treatment	16 Hrs	24 Hrs	40 Hrs	48 Hrs	Day 3	Day 4	Day 15	% Survival
2.5×10^5^	LukS-PV IgG	7/10	7/10	6/10	6/10	6/10	6/10	6/10	60%
2.5×10^5^	Naïve IgG	0/10							0%
9.4×10^4^	LukS-PV IgG	8/10	8/10	8/10	8/10	8/10	8/10	8/10	80%
9.4×10^4^	Naïve IgG	1/10	1/10	1/10	1/10	1/10	1/10	1/10	10%
5.8×10^4^	LukS-PV IgG	10/10	10/10	10/10	10/10	10/10	10/10	10/10	100%
5.8×10^4^	Naïve IgG	4/10	2/10	2/10	2/10	2/10	2/10	2/10	20%
4.3×10^4^	LukS-PV IgG	10/10	10/10	10/10	10/10	10/10	10/10	10/10	100%
4.3×10^4^	Naïve IgG	4/10	3/10	3/10	3/10	3/10	3/10	3/10	30%

### Neutralizing Polyclonal Anti-LukS-PV Antibody Inhibits Oligomerization

**Figure 10:**
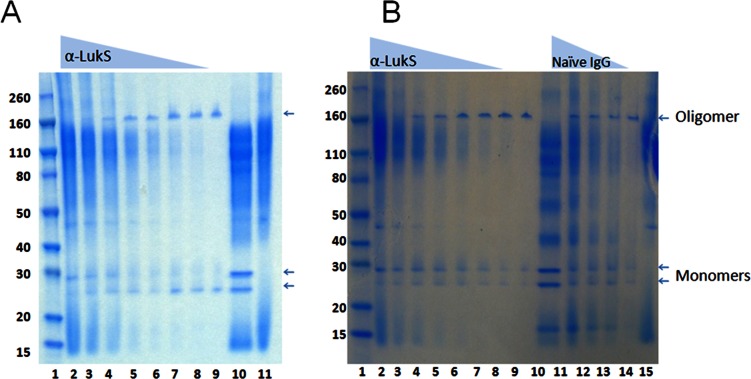
Inhibition of oligomerization by anti LukS-PV polyclonal antibody. ***A)*** Lane1: Marker; lanes 2–8:, 30 µg/ml of LukS-PV was incubated for 30 min at RT with anti-LukS-PV rabbit polyclonal antibodies (pAbs) at 2-fold decreasing concentrations (1.1 mg/ml to 0.017 mg/ml) and then equal concentration of LukF-PV subunit was added; lane 9: LukS+LukF without pAbs; lane10: LukS+LukF+pAbs without MPD; lane 11: pAbs+MPD only. ***B)*** Inhibition of oligomeric band formed by LukS-PV+hlgB by anti-LukS-PV pAb. Lane1: Marker; lanes 2–8∶30 ug/ml of LukS-PV was incubated for 30 min at RT with anti-LukS-PV rabbit polyclonal antibodies (pAbs) at 2-fold decreasing concentrations (1.1 mg/ml to 0.017 mg/ml) then equal concentration of HlgB subunit was added; lane 9: LukS-PV+HlgB without pAbs; lane10: LukS-PV+HlgB+pAbs without MPD; lanes 11–14 LukS-PV+HlgB along with naïve rabbit IgG (1.1 mg/ml to 0.14 mg/ml), and lane 15: rabbit anti-LukS-PV pAbs +MPD only.

We then examined if antibodies to LukS-PV can inhibit homologous and heterologous oligomerization of leukocidins. To this end, the ability of LukS-PV to oligomerize with LukF-PV as well as HlgB was tested in presence of rabbit polyclonal antibodies raised against LukS-PV using the MPD assay described in Materials&Methods section. As shown in ***Figure 10A***, anti-LukS-PV rabbit polyclonal antibody inhibited the oligomerization of wild type LukS-PV/LukF-PV in a dose dependent manner. Similarly, the anti-LukS-PV antibody was also able to inhibit the heterologous oligomerization of LukS-PV with HlgB (***Figure 10B***). These data suggest that vaccine induced antibodies can inhibit both homologous and heterologous oligomerization of the S subunit leukocidins.

### Cross Reactive and Cross-neutralizing Antibody Induced by LukS-Mut9 *in vivo*

**Figure 11:**
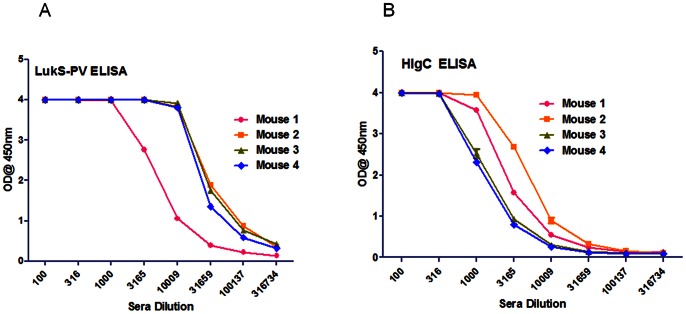
LukS-Mut9 induces cross reactive antibodies. Serum samples after 4 immunizations with LukS-Mut9 were tested for ELISA titers against WT LukS-PV (***A***) and HlgC (***B***).

**Figure 12:**
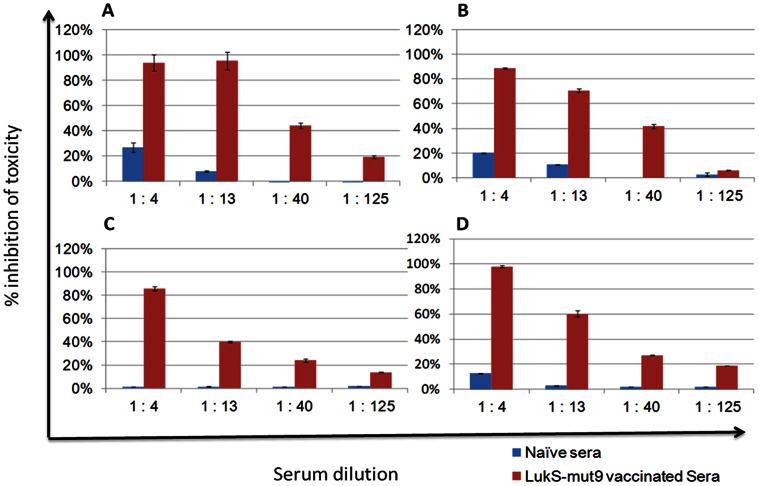
LukS-Mut9 antisera cross neutralize PMN lytic activity induced by other leukocidins. Inhibition by LukS-Mut 9 immunized sera of PMN lytic activity induced by *S. aureus* strain 8325-4 (PVL-neg) supernatant (***A***), USA300 (PVL-pos) Supernatant (***B***), purified PVL (S+F subunits) (***C***), and purified Hlg (B+C subunits) (***D***).

To further examine if the vaccine candidate LukS-Mut9 is able to induce cross neutralizing antibodies, groups of 4 mice were immunized with LukS-PV along with GLA-SE or adjuvant alone and the total IgG and neutralizing titers were determined in sera after four vaccinations. High titers of antibodies to wt LukS-PV **(** ***Figure 11A*** **)** as well as HlgC **(** ***Figure 11B*** **)** could be detected in the sera of all four mice. The serum samples were also tested for the neutralization of PMN lytic activity in *S. aureus* supernatants as well as purified PVL and HlgB/C using the HL60 cells cytotoxicity assay. LukS-Mut9 vaccinated sera effectively neutralized the PMN lytic activity in the culture supernatants from the PVL negative strain NCTC8325-4 (***Figure 12*** **A**) and the PVL positive USA300 (***Figure 12B***), as well as purified PVL (***Figure 12C***) and HlgB/C (***Figure 12D***). These data clearly demonstrate the ability of antibodies induced by LukS-Mut9 to cross protect against non-PVL leukocidins such as Hlg suggesting the potential for broad protection.

## Discussion

Methicillin resistant *S. aureus* (MRSA) invasive disease, defined as isolation of MRSA from an otherwise sterile site, poses a serious challenge to public health around the world. The 2010 CDC surveillance data indicate about 82,000 cases of invasive MRSA in the US with a mortality rate of 14% (http://www.cdc.gov/abcs/reports-findings/survreports/mrsa08.pdf). The incidence rate is highest among dialysis patients with ∼4% of these patients developing invasive MRSA infection every year. According to the same report more than 63% of the CA-MRSA cases and 31% of the HA-MRSA cases were caused by the highly toxigenic and PVL-positive USA300 strain. The remaining cases were caused by the PVL-negative USA100 and USA500. However, these strains produce a number of leukotoxins and hemolysins such as LukED and gamma hemolysin (Hlg) with high level of homology with PVL and overlapping functional characteristics.

Leukocidal toxins (synergohymenotropic toxin) consist of a large group of bicomponent cytotoxins produced by staphylococci. The toxic effect depends on the synergistic action of two proteins. One of them belongs to class F (e.g. LukF-PV, LukF-R, LukF-I, LukM, HlgB, LukD) and the other, to class S (e.g. LukS-PV, LukS-R, LukS-I, HlgA, HlgC, LukE) [14], [31]. These leukocidins exert pore formation on the membranes of human polymorphonuclear leukocytes (PMNs) [32] as well as monocytes and macrophages [12], [33]. In addition to cytolytic activity, leukocidins, primarily PVL, are also strong mediators of inflammatory responses. It has been postulated that PVL intoxicated macrophages orchestrate an excessive inflammatory response in infected tissues [34]. Although PVL is phage encoded, it is striking that it is expressed by all five major pandemic clones. While there has been considerable debate about the role of PVL in CA-MRSA pathogenesis, several reports indicate that PVL affects the severity and outcome of infection in animal models [35], [36], [37], [38]. Epidemiologically, PVL is associated with primary skin infections, such as furunculosis and severe necrotizing pneumonia. Recently we have reported a protective effect of pre-existing antibodies to several *S. aureus* toxins including PVL against sepsis in *S. aureus* bacteremic patients [23].

Collectively, the experimental findings explain the limited contribution of any single member of the leukocidin family to pathogenesis as there is a great degree of redundancy and overlapping functions among the highly similar leukocidins (***Table 3***). While the majority of studies on the role of bicomponent toxins in invasive *S. aureus* disease have been focused on PVL, a number of animal studies suggest a key role for other members of this family. Alonzo *et al* recently reported a key role for LukED in *S. aureus* bloodstream infection in a mouse model [39]. Malachowa *et al* reported an upregulation of the genes for HlgA, B, and C in USA 300 when grown in human blood and defined a role for Hlg in *S. aureus* survival in blood [40]. An isogenic hlgABC-deletion strain (LAC*ΔhlgABC*) had significantly reduced capacity to lyse neutrophils and was attenuated in a mouse sepsis model [40]. Hlg is also shown to play a role in septic arthritis [41] and endophtalmititis [42]. Another novel *S. aureus* leukotoxin, LukGH, has also been recently reported to synergize with PVL to enhance human PMN lysis [43].

**Table 3:** Sequence identity between S and F subunit leukotoxins.

	Class	LukS-PV	LukF-PV
LukS-PV	S	100%	28%
HlgC	S	78%	28%
HlgA	S	69%	26%
LukM	S	68%	26%
LukE	S	68%	26%
HlgB	F	29%	83%
LukD	F	30%	82%

Based on current evidence on the role of various bicomponent leukotoxins in *S. aureus* pathogenesis, we sought to develop attenuated forms of PVL as it represents the most potent member of this family of toxins expressed by major pandemic clones. We hypothesized that mutations can be designed to disrupt the ability of LukS-PV and LukF-PV to oligomerize, a function critically required for cytolysis. Furthermore, we hypothesized that a vaccine based on PVL subunits can provide neutralizing antibodies towards other members of the leukotoxin family. We had previously reported an all-atom model of the macromolecular structure of PVL in its octameric, pre-pore conformation [18]. Using this model we identified several candidate residues for generation of attenuated toxoid subunits. A fully attenuated triple mutant of LukS-PV (LukS-Mut9) was identified consisting of the following mutations: T28F/K97A/S209A. LukS-Mut9 showed no detectable cytotoxicity when combined with wt LukF-PV. In contrast to wt LukS-PV, LukS-Mut9 was unable to form oligomers with the wt forms of either LukF-PV or HlgB. Similarly, a single point mutant of LukF-PV with a K102A mutation (LukF-Mut1) was generated that was highly attenuated when combined with wt LukS-PV. Two additional mutations of LukF-PV (D121A and E147A) also showed partial attenuation, however, to a lesser extent compared to LukF-Mut1. As expected, the combination of LukS-Mut9 and LukF-Mut1 was completely inactive in cytotoxicity assays. Consistent with the partial attenuation phenotype, LukF-Mut1 oligomerization with wt LukS-PV was reduced but not completely abrogated.

Active vaccination studies using the mutant components and their combinations showed significant protection as well as neutralizing antibodies in a mouse model of USA300 sepsis. Both components were able to provide protection and the protection afforded by the combination was only slightly improved but the improvement was not statistically significant. This may relate to the possibility that neutralizing antibodies to either subunit are sufficient to disrupt the oligomerization and consequently the toxicity of PVL. Vaccination with the mutant subunits also resulted in significant reduction of bacterial load in multiple organs. Our findings regarding the efficacy of the two subunits are consistent with a previous report using wild type components as vaccine [35]. The mechanism of the immunity conferred by our mutant subunits is most likely through neutralizing antibodies. Consistent with this notion, passive immunization with polyclonal antibodies to LukS-PV (LukS-PV IgG) provided significant protection from lethal challenge.

An important objective of this study was to demonstrate that vaccination with attenuated PVL subunits can induce neutralizing antibodies against other members of the leukotoxin family. To this end, LukS-PV IgG prevented both homologous oligomerization of PVL S and F subunits as well as heterologous oligomerization of LukS-PV with HlgB. Furthermore, sera from mice vaccinated with LukS-Mut9 showed cross-reactive antibodies to HlgC and neutralized the PMN lytic activity not only of PVL but also purified gamma hemolysin. Importantly, the LukS-Mut 9 immune sera also neutralized the PMN lysis mediated by supernatants of both PVL-positive strain USA300 as well as the PVL-negative strain 8325-4. Similarly our studies indicate that rabbit polyclonal antibodies to LukS-PV neutralize the PMN lytic activity in the supernatants of the *S. aureus* strain Newman which does carry PVL genes (data not shown). These data suggest that LukS-Mut9 can induce neutralizing antibodies with broad reactivity towards other members of the leukotoxin family.

In summary, using a rational, structure-based approach, we have identified mutant forms of both PVL subunits with the potential for cross protective activity against other members of bicomponent pore forming toxins. These mutants are intended to be components of a multivalent, toxin-based vaccine for prophylaxis of invasive *S. aureus* infections. Such a multivalent vaccine may include other toxoids such as alpha hemolysin as well as superantigens. Studies to evaluate the efficacy of the multivalent formulations are currently underway. A toxin-based multivalent vaccine would represent a novel vaccination concept for *S. aureus* intended to prevent clinical invasive disease rather than achieving sterile immunity as the neutralizing antibodies to immune suppressive and tissue destructive toxins may provide a window of opportunity for the host immune system to control the infection.
